# Glucagon-like peptide-1 receptor agonists in total joint arthroplasty: a comprehensive systematic review of what orthopaedic surgeons should know

**DOI:** 10.1186/s42836-026-00375-w

**Published:** 2026-03-23

**Authors:** Omar Abdelaziz, Ziad G. Zayed, Mohamed Abdo Khalafallah, Mohamed A. Hanafy, Abdalla M. Hadhoud, Khaled A. Elmenawi

**Affiliations:** 1 Faculty of Medicine, Alexandria University, Alexandria, 21526 Egypt; 2 College of Human Medicine, Benha University, Benha, 13511 Egypt; 3 Department of Orthopaedic Surgery, Cleveland Clinic, Cleveland, OH 44195 USA

**Keywords:** Total joint arthroplasty, GLP-1 RAs, Periprosthetic joint infection, Readmission rates, Gastrointestinal side effects

## Abstract

**Background:**

The rising prevalence of obesity and type 2 diabetes mellitus (T2DM) among patients undergoing total joint arthroplasty (TJA) presents a significant clinical challenge, increasing the risk of postoperative complications. Glucagon-like peptide-1 receptor agonists (GLP-1 RAs) have emerged as a potential perioperative optimization strategy, but their impact on TJA outcomes remains debated. This systematic review was conducted to synthesize the evidence on the risks and benefits of GLP-1 RA use in adult patients undergoing primary TJA—specifically, total hip arthroplasty (THA), total knee arthroplasty (TKA), and total shoulder arthroplasty (TSA).

**Methods:**

A systematic review was conducted in accordance with the Preferred Reporting Items for Systematic Reviews and Meta-Analyses (PRISMA) guidelines. The PubMed, Scopus, and Web of Science databases were searched from their inception to June 16, 2025, for studies comparing postoperative outcomes in adult patients undergoing primary total joint arthroplasty (TJA) and using GLP-1 RAs versus a control group. Data on study characteristics, patient demographics, and postoperative outcomes were extracted. Due to significant heterogeneity and overlap in data sources, a narrative synthesis rather than meta-analysis of the findings was conducted. Study quality was assessed using the Newcastle–Ottawa Scale (NOS).

**Results:**

Fifteen retrospective cohort studies involving an aggregate total of 318,143 patients (114,365 THA, 125,505 TKA, 78,273 TSA) were included, with 56,132 receiving GLP-1 RAs. In THA and TKA, GLP-1 RAs use was associated with a reduced risk of periprosthetic joint infection (PJI) (e.g., 1.6% vs. 2.9% at 2 years for THA) and lower 90-day readmission rates (e.g., 1.1% vs. 2.0% for TKA and 1.6% vs. 2.0% for THA). When analyzed by exposure timing, the reduced PJI risk was most consistent in studies that defined GLP-1 RA use in the immediate perioperative period. Reported mean length of stay (LOS) was generally similar or slightly shorter among GLP-1 RA users compared to controls. Multiple studies reported either a reduction or no significant difference in the risk of 90-day emergency department visits. The short-term revision rates and dislocations were infrequent and did not differ significantly between groups in most of the included studies. In the TSA, evidence was inconsistent, with reduced odds of 90-day surgical site infection (SSI) (OR 0.25) in one study; however, no clear trend was observed. Gastrointestinal side effects and conflicting systemic risks were noted across procedures.

**Conclusion:**

Current observational data suggest that perioperative GLP-1 RA use in patients undergoing total hip or knee arthroplasty is not associated with a consistent increase in short-term revision rates and may be associated with a reduced risk of postoperative infection. Evidence regarding TSA remains inconclusive. However, given the retrospective nature of the evidence, substantial overlap in data sources, heterogeneity in exposure definitions, and short-term follow-up, these findings should be considered hypothesis-generating. Future prospective, randomized controlled trials with standardized exposure definitions and longer follow-up are required to confirm these associations and establish causality.

**Supplementary Information:**

The online version contains supplementary material available at 10.1186/s42836-026-00375-w.

## Introduction

The demand for total joint arthroplasty (TJA) is escalating globally, driven by an aging population and the rising prevalence of debilitating joint disease [1, 2]. However, the success of these procedures is increasingly challenged by the complex medical profiles of the patient population [3, 4]. The concurrent rising rates of obesity and type 2 diabetes mellitus (T2DM) are particularly concerning as these conditions are highly prevalent among candidates for joint replacement and are well-established predictors of adverse postoperative outcomes [4, 5]. Patients with obesity and T2DM face a heightened risk of complications ranging from surgical site infections (SSI) and delayed wound healing to venous thromboembolism (VTE) and higher rates of revision surgery, leading to increased patient morbidity and substantial healthcare costs [4, 5].

Historically, surgeons were challenged with the management of high-risk patients undergoing TJA, where some recommended the delay of the procedure until comorbidities were better controlled [1, 6]. This approach, however, presents its own set of challenges as many patients struggle to achieve and maintain significant weight loss or optimal glycemic control through lifestyle modifications or bariatric surgery [1, 7]. This clinical dilemma has encouraged the search for novel and effective preoperative optimization strategies [7]. In recent years, glucagon-like peptide-1 receptor agonists (GLP-1 RAs) have been proposed as a potential non-surgical option to resolve this issue [1, 7]. They were initially developed for the management of type 2 diabetes mellitus (T2DM). Their profound effects on weight loss have led to their widespread adoption for obesity management [2, 4]. The adoption of GLP-1 RAs in the perioperative period has generated considerable interest and debate [4, 6]. While some studies suggest a protective effect, others have raised concerns about an increased risk of different complications, such as gastrointestinal adverse effects, up to mortality [2, 8].

While previous systematic reviews have examined the perioperative effects of GLP-1 RAs across various surgical specialties, a synthesis focusing specifically on total joint arthroplasty (TJA) and its unique procedure-specific complications and healthcare utilization metrics is lacking. Furthermore, prior syntheses have not critically examined how the variable operational definition of GLP-1 RA use (e.g., active at surgery vs. historical prescription) across database studies may account for the heterogeneous findings. A comprehensive synthesis of the available evidence is therefore necessary to resolve the conflict and determine the risks and benefits of using these agents in adult patients undergoing primary total knee arthroplasty (TKA), total hip arthroplasty (THA), or total shoulder arthroplasty (TSA). This systematic review addresses this gap by asking: What are the risks and benefits of GLP-1 RA use on joint-specific complications (e.g., PJI, SSI, revision), systemic adverse outcomes, and healthcare utilization in adult patients undergoing primary total hip, knee, or shoulder arthroplasty? Furthermore, how might the definition of exposure modify these associations? Through the integration of existing evidence, our goal was to provide a comprehensive, evidence-based summary as the growing adoption of GLP-1 RAs continues.

## Methods

### Search strategy

This systematic review was conducted in accordance with the Preferred Reporting Items for Systematic Reviews and Meta-Analyses (PRISMA) 2020 guidelines [9]. The protocol adhered to the Cochrane Handbook for Systematic Reviews of Interventions and was prospectively registered on PROSPERO (Registration number: CRD420251143459) [10]. Three databases—PubMed, Scopus, and Web of Science—were searched from their inception to June 16, 2025. To ensure comprehensive literature coverage, no restrictions were applied to publication date or study setting during the search. Searches were restricted to published, peer-reviewed comparative studies to synthesize outcomes from real-world clinical practice. Trial registries and grey literature were not searched, as the review’s objective was to analyze comparative effectiveness and safety data from completed studies with reported results, rather than study protocols or unpublished data. However, reference lists of eligible papers and relevant reviews were screened for additional records. The search strategy combined Medical Subject Headings (MeSH) and free-text keywords related to glucagon-like peptide-1 receptor agonists (GLP-1 RAs) and total joint arthroplasty (TJA). The full search strings for each database, which utilized the comprehensive strategy detailed in the main text, are provided in Supplementary Table S1.

### Inclusion and exclusion criteria

Eligible studies were comparative studies published as full-text articles in peer-reviewed journals, comparing postoperative outcomes in adult patients undergoing primary THA, TKA, or TSA who received GLP-1 RAs versus a control group (no GLP-1 RAs or standard care). Studies were required to report at least one of the following outcomes with a minimum follow-up of 3 months: perioperative or postoperative complications (e.g., periprosthetic joint infection [PJI], surgical site infection [SSI], venous thromboembolism [VTE], myocardial infarction [MI], pneumonia, acute kidney injury [AKI], or deep vein thrombosis [DVT]), adverse drug events (e.g., gastrointestinal complications such as nausea, vomiting, diarrhea, or constipation), revision rates, length of stay (LOS), readmission rates, or emergency department (ED) visits. The intervention included any GLP-1 RA (e.g., semaglutide, liraglutide, dulaglutide, exenatide, lixisenatide, tirzepatide, or albiglutide). The operational definition of GLP-1 RA use differed across the included administrative database studies, primarily based on prescription fills within a specified window relative to surgery (e.g., “at the time of surgery,” “within 90 days before surgery,” or “within 1 year preoperatively”). The specific exposure definition for each included study was extracted and is presented in Table 1. For our narrative synthesis, these varying definitions were considered conceptually to reflect either preoperative exposure (documented use only before surgery) or perioperative exposure (use documented in a window spanning the immediate pre- and postoperative period). Exclusion criteria included single-arm studies, conference abstracts without results, secondary studies (e.g., reviews), studies involving revision arthroplasty or non-total joint arthroplasty (TJA) procedures, and studies lacking relevant outcome data. Studies with mixed TJA populations, excluding those with separable THA, TKA, or TSA data, were also excluded. Two authors independently screened records using the Rayyan web application, resolving disagreements by discussion with a third author.

**Table 1:** Study characteristics

Author Year & Country	Joint	Patient Cohort Size (G/C)	Design	Data Source & Cohort Range	Follow-up Duration	GLP-1 Ras	GLP-1 RA Exposure Definition
**TSA**							
Choudhury et al. 2025 USA*** [20]	TSA	505/7749	R	TriNetX 2018–2023	Medical: 3 m Surgical: 2y	Semaglutide Dulaglutide Liraglutide Exenatide Tirzepatide Lixisenatide Albiglutide	Stratified by GLP-1 RA prescription status (no specific window reported)
Elsabbagh et al. 2025 USA [15]	TSA (3 m)	5010/18701** 8481/56086	R	PearlDiver (Mariner) 2010–2022	Medical: 3 m Surgical: 2 y	Dulaglutide Exenatide Liraglutide Lixisenatide Semaglutide	Taking GLP-1 therapy at the time of TSA surgery
	TSA (2y)	3444/12692** 5969/41845					
Lawand et al. 2025 USA [23]	TSA	1259/1259* 1259/107067	R	TriNetX 2012–2023	Medical: 3 m Surgical: 2y	Semaglutide Liraglutide	Taken semaglutide or liraglutide within 3 months prior to TSA
Seddio et al. 2025 USA [26]	TSA	632/2302**	R	PearlDiver (Mariner) 2010–2022	3 m	Semaglutide	Use of semaglutide within 1 year before TSA
**THA**							
Kim et al. 2024 USA [18]	THA	771/3084**	R	PearlDiver (Mariner) 2010–2022	Medical: 3 m Surgical: 2y	Exenatide Semaglutide Dulaglutide Liraglutide Exenatide Microspheres	Prescription filled for ≥ 3 months before and after TKA
Magaldi et al. 2024 USA [13]	THA	66/126**	R	Institutional Database 2016–2022	HOOS-JR: pre-op 12wk 6 m 2y post-op	Semaglutide Liraglutide Dulaglutide Exenatide	Presence of a GLP-1 RA on the preoperative medication list
Magruder et al. 2024 USA [16]	THA	1653/7812**	R	PearlDiver (National Claims) 2010–2021	Medical: 3 m Surgical: 2 y	Semaglutide	Prescription for semaglutide at the time of THA
Verhey et al. 2025 USA [25]	THA	5345/5345* 10,891/839715	R	PearlDiver (Mariner) 2010–2022	Medical: 3 m Surgical: 2 y	Liraglutide, Semaglutide, Dulaglutide, Exenatide, or Lixisenatide	Taking a GLP-1 RA between 1 year prior to and 2 years after THA
**TKA**							
Kim et al. 2025 USA [12]	TKA	2975/2975*	R	PearlDiver (Mariner) 2010–2020	Medical: 3 m Surgical: 2y	Exenatide Semaglutide Liraglutide Dulaglutide Albiglutide	Prescription filled for ≥ 3 months before and after THA
Magruder et al. 2023 USA [24]	TKA	7051/34524**	R	PearlDiver 2010–2021	Medical: 3 m Surgical: 2y	Semaglutide	Active prescription for semaglutide at the time of TKA
Heo et al. 2024 USA [17]	TKA	2388/2388* 2388/26117	R	IBM MarketScan/Medicare Supplemental 2016–2021	Medical: 3 m Surgical: 1 y		≥ 3 prescription fills or a ≥ 90-day supply within 6 months preoperatively
Katzman et al. 2025 USA [19]	TKA	865/8650** 865/12886	R	Institutional Database 2012–2023	GLP-1RA: 2.2y Control: 2.9y	Semaglutide Liraglutide Dulaglutide Exenatide Tirzepatide Lixisenatide Albiglutide	Active use within 6 months pre-op and continued use within 3 months post-op
**THA/TKA**							
Baum et al. 2024 USA [14]	THA	667/83587	R	PearlDiver (Mariner) 2010–2022	3 m	Adlyxin Bydureon Byetta Ozempic Rybelsus Saxenda Trulicity Victoza Wegovy	Active prescription for any GLP-1 RA within 90 days prior to surgery
	TKA	1876/150099					
Buddhiraju et al. 2024 USA [22]	THA	1044/1044* 1044/268,504	R	TriNetX 2005––2023	3 m		Prescription filled between 1 year and 15 days preceding surgery
	TKA	2095/2095* 2095/386356					
Levidy et al. 2025 USA [21]	THA	2244/2244 *	R	TriNetX 2005–2024	Medical: 3 m Surgical: 1 y	Liraglutide Pramlintide Tirzepatide Semaglutide Lixisenatide Dulaglutide	Prescription filled within 1 year preoperatively
	TKA	4700/4700*	R	TriNetX 2005–2024	Medical: 3 m Surgical: 1 y		

^*^1:1 Matched cohort

^**^1: > *1 matched cohort*

^***^Choudhury et al. *(2024) employed inverse propensity score-weighting*

*R: Retrospective, THA: Total Hip Arthroplasty, TKA: Total Knee Arthroplasty, TSA: Total Shoulder Arthroplasty, GLP-1 RA: Glucagon-like peptide-1 receptor agonist, post-op: post-operative, HOOS-JR: Hip disability and Osteoarthritis Outcome Score Joint Replacement*

### Data extraction

Data were extracted independently by two authors using a predefined standardized spreadsheet. Extracted details included study design, sample size, data source, cohort period, patient demographics (age, sex, body mass index [BMI], comorbidities such as diabetes mellitus), intervention details (GLP-1 RA type and exposure definition), comparator, and outcomes (perioperative/postoperative complications, adverse drug events, revision rates, LOS, readmission rates, and ED visits). For continuous outcomes (e.g., LOS), means and standard deviations were recorded when available; medians with ranges were noted if means were unavailable. Discrepancies between authors were resolved by consensus with a third author.

### Quality assessment

The Newcastle–Ottawa Scale (NOS) [11] was used to assess the quality of included retrospective cohort studies across three domains: selection, comparability, and outcome (nine items, scored 1–9). Two authors independently evaluated each study, assigning scores based on cohort selection (e.g., representativeness, exposure ascertainment), comparability of groups (e.g., use of propensity score matching), and outcome assessment (e.g., follow-up duration, adequacy of outcome reporting). Disagreements were resolved by consultation with a third experienced author.

### Data synthesis

A quantitative meta-analysis was deemed inappropriate due to significant clinical and methodological heterogeneity across studies, including variations in patient populations, GLP-1 RA agents, outcome definitions, and follow-up duration. Crucially, the substantial risk of patient cohort overlap across studies using the same large administrative databases (e.g., TriNetX, PearlDiver) precluded the reliable pooling of data. Therefore, a narrative synthesis was conducted. To ensure transparency and mitigate the risk of double-counting patients from overlapping data sources, we implemented the following approach: (1) the primary database and cohort period for each study were clearly documented in Table 1; (2) when multiple studies utilized the same database with overlapping timeframes, this overlap was explicitly acknowledged in the narrative results, and their quantitative findings were not combined; and (3) the synthesis prioritizes comparing the direction and consistency of findings across independent studies, rather than relying on the aggregated sample size, which may be inflated by overlap. Findings were organized by procedure (THA, TKA, TSA) and then by outcome category (e.g., healthcare utilization, implant-related complications, systemic adverse events). For each outcome, results are presented descriptively, highlighting the direction of the effect (e.g., reduced, increased, neutral) and the approximate magnitude (e.g., odds ratios, risk differences) as reported in the primary studies. The aggregated total sample size is provided for context, but it is interpreted with caution due to acknowledged overlap.

## Results

### Screening process and study selection

**Fig. 1:**
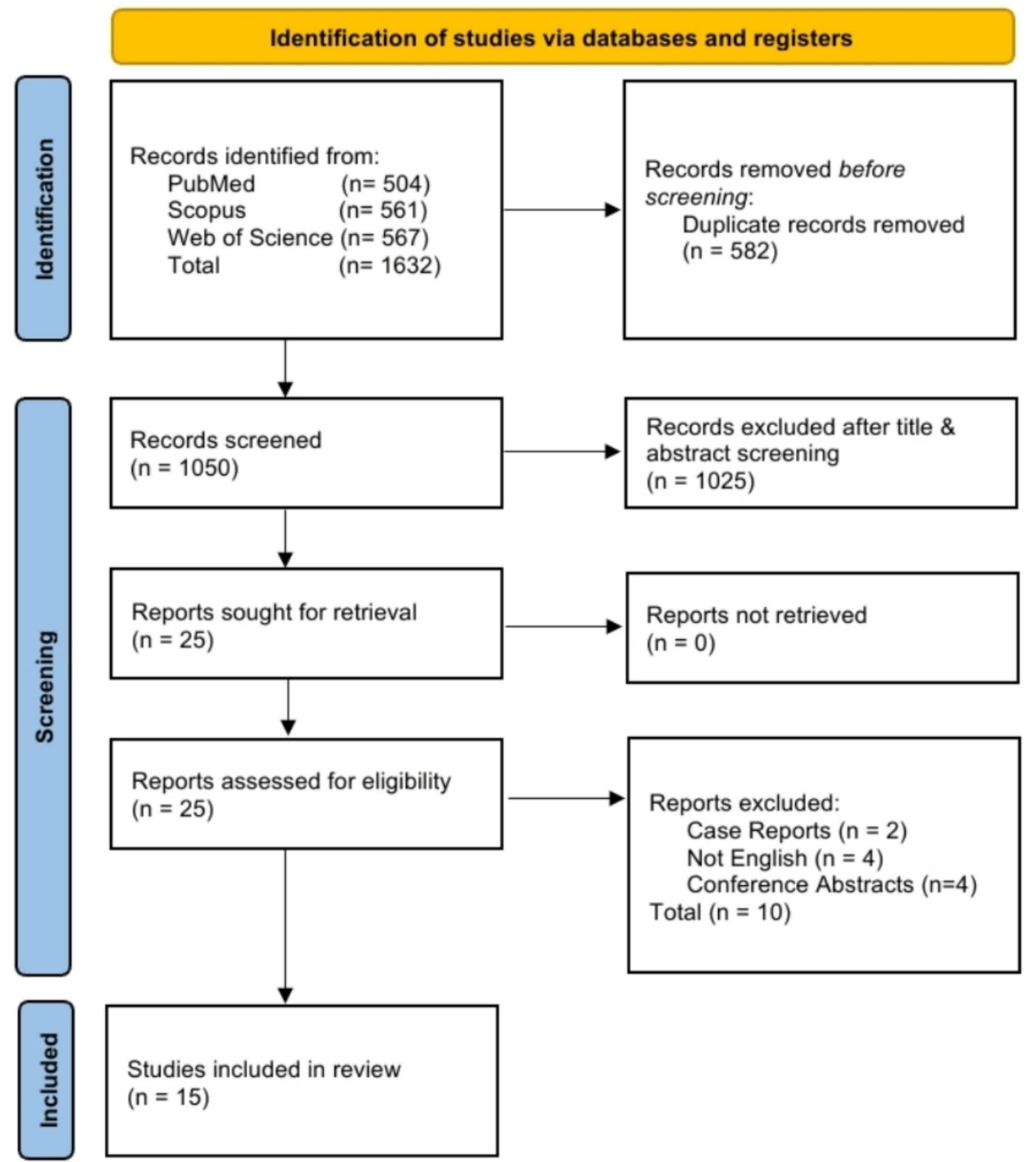
PRISMA flow diagram

The literature search identified 1,632 records. After removing 582 duplicates, 1,050 unique records were screened, of which 1,025 were excluded. The full texts of the remaining 25 articles were reviewed, and 15 studies [12–26] ultimately met the inclusion criteria. The PRISMA flow diagram detailing the study selection process is presented in Fig. 1.

### Patient and study characteristics

The 15 included retrospective cohort studies [12–26] reported on an aggregate of 318,143 patients (114,365 THA, 125,505 TKA, 78,273 TSA), with 56,132 patients prescribed a GLP-1 RA. This total likely overestimates the number of unique patients due to overlap across large U.S. administrative databases (e.g., TriNetX, PearlDiver). A critical point of heterogeneity was the operational definition of GLP-1 RA exposure, which varied significantly across studies. (Table 1) Definitions ranged from broad historical prescriptions (e.g., any prescription within 1 year before surgery) to strict perioperative criteria (e.g., active prescription at the time of surgery, or for a minimum period spanning 3 months before and after the procedure). Thirteen studies used propensity score matching (ratios 1:1 to 10:1), one used inverse probability weighting [20], and one used an unmatched cohort design [14]. All studies were U.S.-based and published between 2023 and 2025.

Most studies included THA/TKA patients (*n* = 11); four focused on TSA [15, 20, 23, 26]. Follow-up averaged 18 months, typically reporting 90-day and/or 2-year outcomes. Most studies assessed various GLP-1 RAs, with three focusing exclusively on semaglutide [16, 24, 26]. The mean baseline age (64.6 years) and BMI (35 kg/m^2^) were similar across groups. (Table 1).

Comorbidity burden, assessed via the Charlson Comorbidity Index (CCI) or Elixhauser Comorbidity Index (ECI), and preoperative HbA1c (range: 6.3–6.5) were well-balanced. Osteoarthritis was the primary surgical indication. A total of 108,107 patients had diabetes, of whom 22,495 (21%) received a GLP-1 RA. Six studies enrolled exclusively diabetic patients [12, 15, 17, 20, 21, 23], one enrolled only non-diabetic patients [25], and the others enrolled mixed cohorts. Patients in both groups commonly used other anti-diabetic medications (e.g., Metformin, Insulin). (Tables 2 & 3).

**Table 2:** Patients’ characteristics

Author Year & Country	Mean Age		Male (*n*/total %)		Pre-op Score (Index)		Indication		Anti-diabetic Medications (*n*)	
	**G**	**C**	**G**	**C**	**G**	**C**	**G**	**C**	**G**	**C**
**TSA**										
Choudhury et al. 2025 USA*	67.5 ± 7.6	70.4 ± 8.1	221/505 (43.8%)	3343/7749 (43.1%)	3.1 ± 2.7 (CCI)	2.9 ± 2.8 (CCI)			Metformin: 412 Insulin: 420	Metformin: 4700 Insulin: 5429
Elsabbagh et al. 2025 USA (3 m)	68.2 ± 6.8	68.3 ± 6.7	2258/5010 (45%)	8419/18701 (45%)	2.7 ± 1.9 (CCI)	2.6 ± 1.8 (CCI)	1ry OA		Metformin: 3471 Insulin: 899	Metformin: 12,880 Insulin: 2859
Elsabbagh et al. 2025 USA (2y)	67.9 ± 6.5	67.9 ± 6.5	1549/3444 (45%)	5674/12692 (44.7%)	2.6 ± 1.8 (CCI)	2.5 ± 1.7 (CCI)			Metformin: 2224 Insulin: 508	Metformin: 8122 Insulin: 1512
Lawand et al. 2025 USA	66.5 ± 8.7	66.3 ± 9.4	500/1259 (39.7%)	474/1259 (37.7%)						
Seddio et al. 2025 USA	65.0 ± 6.3	65.0 ± 6.1	287/632 (45.4%)	1040/2302 (45.2%)	8.0 ± 3.1 (ECI)	8.0 ± 3.0 (ECI)	Advanced Glenohumeral Degeneration		Insulin: 380 Metformin: 568	Insulin: 1342 Metformin: 2081
**THA**										
Buddhiraju et al. 2024 (THA) USA	63.3 (53.7–72.9)	63.5 (53.6–73.4)	442/1044 (42.3%)	427/1044 (40.9%)						
Kim et al. 2024 USA	62.1 ± 8.3	62.1 ± 8.3	364/771 (47.2%)	1453/3084 (47.1%)	3.3 ± 2.2 (CCI)	3.3 ± 2.2 (CCI)	1ry OA			
Magaldi et al. 2024 USA	67.9 ± 8.8	66.3 ± 7.0	38/66 (57.6%)	73/126 (57.9%)						
Magruder et al. 2024 USA			866/1653 (52.4%)	4076/7812 (52.2%)			1ry OA		Metformin: 1540	Metformin: 7313
Verhey et al. 2025 USA	57 ± 8.6	57 ± 8.5	1657/5345 (31.0%)	1657/5345 (31.0%)	2.7 ± 1.8 (ECI)	2.7 ± 1.8 (ECI)				
**TKA**										
Kim et al. 2025 USA	62.2 ± 7.6	62.2 ± 7.6	989/2975 (33.2%)	986/2975 (33.1%)	3.3 ± 2.3 (CCI)	3.3 ± 2.3 (CCI)	1ry OA			
Magruder et al. 2023 USA			2720/7051 (38.6%)	13,265/34524 (38.4%)			1ry OA		Glipizide: 2032 Glimepiride: 2128 Sitagliptin: 2103 Pioglitazone: 1568 Empagliflozin: 1522 Metformin:6354	Glipizide: 6483 Glimepiride: 5876 Sitagliptin: 5106 Pioglitazone: 4224 Empagliflozin: 1054 Metformin:31,206
Buddhiraju et al. 2024 (TKA) USA	64.1 (55.8–72.4)	64.2 (55.8–72.6)	693/2095 (33.1%)	683/2095 (32.6%)						
Heo et al. 2024 USA	61.2 (40–88)	61.0 (32–88)	1013/2388 (42.4%)	1040/2388 (43.5%)			1ry OA		Insulin: 525 other: 1834	Insulin: 491 other: 1878
Katzman et al. 2025 USA	64 (30–97)	64 (21–94)	294/865 (34%)	2911/8650 (33.7%)	4.4 (0–18) (CCI)	3.2 (0–18) (CCI)	1ry OA: 848 Post-traumatic: 13 Inflammatory: 2 ON: 2	1ry OA: 8407 Post-traumatic: 170 Inflammatory: 57 ON: 16	Insulin: 563 Metformin: 591	Insulin: 3074 Metformin: 3696

*Levidy et al. 2025 and Baum et al. 2024 studies didn’t report these specific values for demographics*

For studies employing propensity score matching (PSM)/inverse probability weighting (IPW), values represent the post-matching cohort unless otherwise specified in the source study. For studies without PSM or IPSW, values represent the raw, unmatched cohorts*

OA: Osteoarthritis, ON: Osteonecrosis, Pre-op: Pre-operative, SD: Standard Deviation, CCI: Charlson Comorbidity Index, ECI: Elixhauser Comorbidity Index, G: Glucagon-like peptide-1, C: Control, THA: Total Hip Arthroplasty, TKA: Total Knee Arthroplasty, TSA: Total Shoulder Arthroplasty, y: Year, m: Month, USA: United States of America

**Table 3:** Patients comorbidities

Author Year & Country	Diabetes (*n*/total %)		Preoperative HbA1c (Mean ± SD)		HTN (*n*/total %)		Obesity (*n*/total %)		BMI (Mean ± SD)	
	**G**	**C**	**G**	**C**	**G**	**C**	**G**	**C**	**G**	**C**
**TSA**										
Choudhury et al. 2025 USA	505/505 (100%)	7749/7749 (100%)							35.36 ± 6.33	32.94 ± 6.63
Elsabbagh et al. 2025 USA (3 m)	5010/5010 (100%)	18,701/18701 (100%)			4511/5010 (90.0%)	16,521/18701 (88.3%)	2613/5010 (52.2%)	8165/18701 (43.7%)		
Elsabbagh et al. 2025 USA (2y)	3444/3444 (100%)	12,692/12692 (100%)			3031/3444 (88.0%)	10,959/12692 (86.3%)	1593/3444 (46.3%)	4822/12692 (38.0%)		
Lawand et al. 2025 USA	1073/1259 (85.2%)	1071/1259 (85.2%)			1138/1259 (90.4%)	1129/1259 (89.7%)	974/1259 (77.4%)	969/1259 (77.0%)	35.5 ± 6.5	34.1 ± 6.6
Seddio et al. 2025 USA	632/632 (100%)	2302/2302 (100%)								
**THA**										
Buddhiraju et al. 2024 (THA) USA	723/1044 (69.3%)	713/1044 (68.3%)	6.5 (5.3–7.7)	6.5 (5.1–7.9)	838/1044 (80.3%)	853/1044 (81.7%)	734/1044 (70.3%)	764/1044 (73.2%)		
Kim et al. 2024 USA	403/771 (52.3%)	1612/3084 (52.3%)					771/771 (100%)	3084/3084 (100%)		
Magaldi et al. 2024 USA									35.0 ± 4.6	34.7 ± 4.5
Magruder et al. 2024 USA	1653/1653 (100%)	7812/7812 (100%)					1404/1653 (84.9%)	6655/7812 (85.2%)		
Verhey et al. 2025 USA	0/5345 (0%)	0/5345 (0%)					3647/5345 (68.2%)	3650/5345 (68.3%)		
Levidy et al. 2025 (THA) USA	2244/2244 (100%)	2244/2244 (100%)								
**TKA**										
Kim et al. 2025 USA	1571/2975 (52.8%)	1571/2975 (52.8%)					2975/2975 (100%)	2975/2975 (100%)		
Magruder et al. 2023 USA	7051/7051 (100%)	34,524/34524 (100%)					6081/7051 (86.2%)	29,844/34524 (86.4%)		
Buddhiraju et al.2024 (TKA) USA	1439/2095 (68.7%)	1433/2095 (68.4%)	6.5 (5.2–7.8)	6.4 (5.2–7.6)			1439/2095 (68.8%) *	1476/2095 (70.6%)*		
Heo et al. 2024 USA	2388/2388 (100%)	2388/2388 (100%)								
Katzman et al. 2025 USA	644/865 (74.4%)	3244/8650 (37.5%)	6.5 (4.3–11.3)	6.3 (3.4–15.2)						
Levidy et al. 2025 (TKA) USA	4700/4700 (100%)	4700/4700 (100%)								

Baum et al. (2024) study didn’t report these specific proportions for co-morbidities

^*^Buddhiraju et al. (2024) report a combined total of all patients who had a diagnosis of either overweight, obesity, or hyperalimentation

SD: Standard Deviation, BMI: Body Mass Index, G: Glucagon-like peptide-1, C: Control, THA: Total Hip Arthroplasty, TKA: Total Knee Arthroplasty, TSA: Total Shoulder Arthroplasty, y: Year, m: Month, USA: United States of America

### Summary of quality assessment

Generally, studies [12–26] have shown good performance across the selection, comparability, and outcome domains. Aside from Baum et al. [14], which showed moderate quality, the overall body of evidence was predominantly of high quality, with scores ranging from eight to nine. It is important to note that while the NOS assesses specific methodological criteria, a high score does not eliminate the fundamental limitations inherent to observational database studies, such as residual confounding or exposure misclassification. Points were reduced in three studies (Baum et al. [14], Buddhiraju et al. [22], Seddio et al. [26]) due to short-term follow-up, while in one study (Baum et al. [14]), it was due to the omission of propensity score matching. (Table S2).

### Healthcare utilization outcomes

A) Total Hip and Knee Arthroplasty (THA/TKA)
  The length of stay (LOS) was generally similar between the groups. In studies reporting a difference, LOS was slightly shorter for GLP-1 RA users, particularly in high-BMI populations (e.g., 2.7 vs. 2.9 days for TKA [12]; 2.2 vs. 3.1 days for THA [18]). The 90-day readmission rates were low and not consistently different, with reported rates such as 1.6% vs. 2.0% in THA and 1.1% vs. 2.0% in TKA (GLP-1 RA vs. control) [22]. Evidence on 90-day emergency department (ED) visits was mixed; one study of non-diabetic THA patients reported reduced odds (OR 0.81) [25], while another in TKA reported higher rates (5.9% vs. 4.0%) [19]. (See Table S3 for full details).
B) Total Shoulder Arthroplasty (TSA)
  Data on healthcare utilization in TSA were limited. One study reported increased odds of 90-day readmission rates (OR 1.6) [23], while another found significantly fewer 90-day ED visits among GLP-1 RA users (OR 0.37) [26]. LOS was not a commonly reported outcome for TSA. (See Table S3 for full details).

### Implant-related and local complications

A) Total Hip and Knee Arthroplasty (THA/TKA)
  A different pattern appeared when evaluating the results based on the timing of GLP-1 RA exposure. (Table 1) Studies that defined exposure in the active perioperative period—either at the time of surgery or spanning the weeks immediately before and after—consistently reported a reduced risk of periprosthetic joint infection (PJI). For instance, in THA, Magruder et al. [16] (defining use at the time of surgery) reported 2-year PJI rates of 1.6% vs. 2.9%. Similarly, Kim et al. [18] (defining use for ≥ 3 months before and after surgery) found 90-day PJI rates of 1.6% vs. 3.2% in a morbidly obese cohort. In TKA, Kim et al. [12] (exact perioperative definition) reported 90-day PJI rates of 1.0% vs. 1.8%. In contrast, the single THA/TKA study using a less specific, longer-term preoperative definition (Buddhiraju et al. [22]: prescription within 1 year preoperatively) found a more modest reduction in 90-day SSI risk (2.1% vs. 3.6% in THA), and no significant difference in TKA (1.1% vs. 1.1%). Rates of short-term revisions and mechanical complications were infrequent and did not differ significantly between groups across all exposure definitions. (See Table S4 & S4 for full details)
B) Total Shoulder Arthroplasty (TSA)
  The limited evidence in TSA was inconsistent and reflected differing exposure windows. Seddio et al. [26] (use within 1 year before surgery) reported significantly lower odds of 90-day surgical site infection (SSI) (OR 0.25). Conversely, Choudhury et al. [20] (only preoperative use of GLP-1 RAs) found no significant difference in 2-year PJI/SSI. (See Tables S3 & S4 for full details).

### Systemic complications and adverse events

A) Total Hip and Knee Arthroplasty (THA/TKA)
  The systemic safety profile was mixed. Notably, the use of a strict perioperative exposure definition was associated with higher odds of 90-day myocardial infarction (OR 1.49) and pneumonia (OR 1.67) in TKA in one study [24], a signal not observed in studies with preoperative-only definitions. Conversely, a separate THA study using a similar perioperative definition reported reduced odds of 90-day acute kidney injury (OR 0.69) and hypoglycemic events (OR 0.45) [16]. For other outcomes, including 90-day venous thromboembolism (VTE) and mortality, no consistent pattern or significant difference was observed in most studies. As expected, 90-day gastrointestinal (GI) side effects were more common among GLP-1 RA users regardless of the exposure definition, aligning with the established drug class profile. (See Tables S5–S7 for full details).
B) Total Shoulder Arthroplasty (TSA)
  The contradictory systemic findings in TSA may be linked to the differing exposure windows. The study reporting reduced risks for 90-day VTE (OR 0.36), cardiac events (OR 0.32), and pneumonia (OR 0.25) used a broad, preoperative definition (within 1 year) [26]. The study reporting increased risks for 90-day DVT (OR 3.0), MI (OR 2.8), and pneumonia (OR 2.2) used a narrow, preoperative definition (within 3 months) [23]. This contrast underscores that the net systemic effect in TSA may critically depend on the proximity of drug use to surgery, a relationship obscured when exposure is not precisely defined. One study reported significantly reduced 90-day mortality (OR 0.077) [20]. Gastrointestinal complications were not reported as an outcome in the TSA studies. (See Tables S5–S7 for full details).

## Discussion

This systematic review compiles evidence from 15 retrospective cohort studies involving an aggregate total of 318,143 patients who underwent primary total hip arthroplasty (THA), total knee arthroplasty (TKA), or total shoulder arthroplasty (TSA). A narrative synthesis of this observational data suggests that perioperative GLP-1 RA use is not associated with a clear, consistent signal of increased major adverse events in the studied populations. Furthermore, the data suggest a possible link to a decreased likelihood of certain postoperative complications, particularly local infections within high-risk cohorts undergoing THA and TKA. Nevertheless, considerable discrepancies persist, especially regarding systemic adverse events and the anticipated gastrointestinal (GI) side effects. A key finding from our synthesis is that the timing of GLP-1 RA exposure in relation to the surgical procedure proved to be a crucial determinant, potentially accounting for the variability observed in reported outcomes, specifically concerning infection risk and systemic complications. Collectively, these findings generate essential hypotheses and underscore the need for prospective validation by studies with standardized exposure definitions.

### Healthcare utilization outcomes

Based on the available observational data, the use of perioperative GLP-1 RA was not associated with a consistent negative impact on short-term healthcare utilization metrics. For THA and TKA, the length of stay (LOS) was similar or slightly shorter among GLP-1 RA users compared to controls, with reported averages of 2.2 days versus 3.1 days in THA and 2.7 days versus 2.9 days in TKA [12, 18]. This aligns with the known pharmacodynamics of GLP-1 RAs, which are characterized by delayed gastric emptying but generally do not prolong recovery times in orthopaedic settings [27]. The 90-day readmission rates were also low and not consistently different among the groups [12, 22]. The use of emergency department (ED) services was inconsistent; however, some data suggested a possible benefit, with several studies favoring GLP-1 RA users, especially within the THA group [22, 25, 26]. This decrease may indicate improved glycemic control or enhancements in mobility and self-care due to weight loss, thereby reducing the necessity for acute postoperative care [5, 28, 29]. For TSA, data on healthcare utilization were limited. However, one study found significantly fewer 90-day ED visits (OR 0.37) and another one reported an increased odds of 90-day readmissions (OR 1.6) [23, 26].

### Implant-related and local complications

One of the most notable associations in the included studies was a signal suggesting a potentially reduced risk of local complications, particularly surgical site infections (SSI) and periprosthetic joint infections (PJI), in patients undergoing THA and TKA who received treatment with GLP-1 RAs. Our narrative synthesis revealed that the apparent protective signal was most consistent and pronounced in studies that defined GLP-1 RA exposure in the immediate perioperative period (i.e., at the time of surgery or spanning weeks before and after), compared to those using longer-term preoperative definitions. In high-risk populations—such as those with morbid obesity or diabetes—several studies reported significantly lower rates of PJI/SSI [16, 18, 21, 24]. For example, in diabetic patients undergoing THA, the 2-year PJI rates were 1.6% compared to 2.9% [16], and similar patterns were noted in TKA groups [12, 21, 24]. This association may be mediated by the acute anti-inflammatory and glycemic-stabilizing effects of GLP-1 RAs during wound healing, rather than chronic metabolic benefits from long-term use [28–30]. Furthermore, the reduced incidence of hematoma in THA (0% vs. 1.3%) suggests a possible hemostatic advantage, which may be associated with decreased inflammation [18]. These findings are consistent with a recent meta-analysis suggesting that GLP-1 RA use was associated with reduced postoperative complications, including lower rates of periprosthetic joint infection (PJI), following total knee or hip arthroplasty [31].

The results regarding implant survival, which involve revision surgeries and dislocations, were infrequent and comparable across the different groups within the available short follow-up period. The revision rates ranged from 0.6% to 4.0%, showing no association with an increased early risk among GLP-1 RA users [22, 25]. Similarly, the dislocation rates were low, ranging from 0.8% to less than 2.3% [18, 25]. These findings may be reassuring for short-term implant stability, particularly given the hypothetical concerns about potential GLP-1 RA-related sarcopenia or alterations in bone density that may affect implant survivorship [32]. However, the predominantly short-term follow-up, averaging 18 months, is a critical limitation. Consequently, although the available data do not indicate an increased short-term revision risk, they are insufficient to conclude on long-term safety regarding implant fixation and survival [16, 24]. The neutral short-term revision risk observed aligns with recent meta-analyses indicating neutral fracture risks among diabetic populations on GLP-1 RAs [33]. Nevertheless, prospective studies with extended follow-up are necessary to definitively address this clinical gap.

In contrast, the results on TSA were more diverse, with one study showing a significant decrease in 90-day surgical site infection (SSI) odds (OR 0.25) [26], while another one found no significant differences or trends towards SSI [20]. The inconsistency in TSA findings may also relate to differing exposure definitions, further highlighting how variable methodology can obscure a clear signal. Periprosthetic fractures were rare and showed neutral outcomes across all procedures, suggesting that GLP-1 RA was not associated with harm to bone health [34]. This discrepancy may reflect fundamental differences between the procedures. Periprosthetic joint infection (PJI) in weight-bearing joints like the hip and knee is strongly linked to obesity and metabolic syndrome, conditions that GLP-1 RAs directly target. In contrast, the infection risk in non-weight-bearing shoulder arthroplasty is less clearly tied to these systemic metabolic factors, which may explain why a consistent benefit from GLP-1 RAs is not observed in TSA [4].

### Systemic complications and adverse events

Systemic complications showed a wide range of outcomes, with notable inconsistencies across various studies and procedures. In THA and TKA, the evidence for systemic cardiopulmonary, renal, and thromboembolic events was conflicting, without a clear indication of protective or harmful effects. For instance, one study reported potential advantages, such as lower transfusion requirements (OR 0.53 for non-diabetics) and reduced blood loss anemia (OR 0.57) [25]. Conversely, other studies indicated mixed risks: one in TKA found higher odds of 90-day myocardial infarction (OR 1.49) and pneumonia (OR 1.67) [24]. At the same time, another reported lower rates of 90-day acute kidney injury (3.4% vs. 4.0%) [12]. The study reporting increased cardiopulmonary risks employed a strict perioperative exposure definition, suggesting the timing of drug use may influence risk. In contrast, gastrointestinal side effects were consistently more frequent among GLP-1 RA users, with increased rates of nausea/vomiting (18.2% compared to 6.0%) and diarrhea, aligning with the known effects of GLP-1 RAs on gastric motility [13, 27]. Considering this mixed profile, careful perioperative management to minimize gastrointestinal symptoms is essential, along with current anesthesiology guidelines [35].

In TSA, the findings were highly contradictory: one study indicated lower risks of 90-day venous thromboembolism (OR 0.36), cardiac events (OR 0.32), and pneumonia (0.25) [26], while another showed higher risks (DVT OR 3.0; MI OR 2.8; pneumonia OR 2.2) [23]. This variation likely stems from more than just differences in cohort characteristics, follow-up duration, or the use of anticoagulants [36]. The opposing perioperative effects of GLP-1 RAs may lead to different outcomes, and the systemic benefits of the medications, such as reduced inflammation and enhanced endothelial function, may explain the observed decreased risks [37]. However, in specific patient subgroups, changes in body weight and fluid balance induced by GLP-1 RA therapy could be less well tolerated—particularly in individuals with lower baseline BMI or pre-existing cardiac dysfunction—potentially contributing to transient physiological stress and explaining conflicting results [38]. Importantly, these biological effects may be linked to how studies define exposure windows. The study showing reduced risks considered use within 1 year before surgery, likely representing long-term stable users [26]. Conversely, the study reporting increased risks focused on use within 3 months before surgery, potentially capturing patients experiencing acute physiological changes [23]. This contrast underscores that the overall systemic effect may depend on the definition of drug use relative to surgery. Given this profound inconsistency, coupled with the limited number of TSA studies, the evidence is currently insufficient to support any reliable conclusion regarding the systemic safety or risk profile of GLP-1 RAs in shoulder arthroplasty. Isolated signals, such as a reduction in mortality (OR 0.077) [20], are intriguing but require validation in larger, prospective cohorts. The absence of data on gastrointestinal complications in TSA further limits a comprehensive assessment of the condition.

### Implications and future directions

For the orthopaedic surgeons, the synthesis of current observational data suggests that, in patients undergoing total hip or knee arthroplasty, perioperative GLP-1 RA use has not been associated with a clear signal of increased short-term surgical risk in the available studies. A consistent finding across several studies is a potential association with a reduced risk of periprosthetic joint infection, particularly in subgroups with obesity or diabetes. However, this evidence is derived solely from retrospective cohorts with methodological heterogeneity, most notably in the definition of drug exposure. For total shoulder arthroplasty, the evidence is limited and contradictory, offering no interpretable signal. Therefore, while these data are hypothesis-generating and can inform the design of future studies, they are insufficient to guide definitive clinical practice. Clinical decisions regarding perioperative GLP-1 RA management should remain individualized and based on multidisciplinary assessment, considering the known gastrointestinal side-effect profile. Future research must prioritize prospective, controlled designs to establish causality. These studies should employ standardized, clinically relevant definitions of GLP-1 RA exposure (e.g., active perioperative use vs. long-term prescription) to clarify the timing-dependent effects suggested by this review. Additional priorities include extended follow-up to assess long-term implant survivorship, differentiation by procedure and specific GLP-1 RA agent, and the inclusion of patient-reported outcomes.

### Strengths and limitations

This systematic review synthesizes evidence from retrospective cohort studies, many of which used large, nationally representative U.S. databases (e.g., TriNetX, PearlDiver), providing a reasonably broad overview of GLP-1 RA use in total joint arthroplasty. The review also incorporates diverse patient populations, including those with diabetes, obesity, and non-diabetic individuals, offering insight into potential variations in GLP-1 RA effects across different subgroups. A primary methodological contribution of this review is its critical analysis of the GLP-1 RA exposure definition. By systematically extracting and presenting each study’s operational criteria (Table 1) and analyzing outcomes in relation to these differing windows, this synthesis moves beyond merely acknowledging heterogeneity. We demonstrate that variable definitions (e.g., “active at surgery” vs. “prescription within 1 year”) can lead to divergent conclusions, providing a transparent framework for interpreting conflicting evidence, particularly in the context of TSA. This establishes exposure timing as a key consideration for future research.

However, the findings must be interpreted within the context of notable limitations inherent in the available evidence. Primarily, all included studies are retrospective and observational, which means causality cannot be established. A major concern is confounding by indication. Patients prescribed GLP-1 RAs form a selected group who tend to receive more structured metabolic management, closer perioperative monitoring, and may be more health-conscious—factors that independently reduce surgical risk and are challenging to fully measure or adjust for. Although propensity matching addressed many known confounders, residual confounding from unmeasured variables (e.g., nutritional status, physical activity, socioeconomic factors) is likely to remain. Additionally, methodological limitations of database studies are relevant. Risks of exposure and outcome misclassification exist; a filled prescription does not confirm perioperative adherence, and coding inaccuracies can influence the reported complications. While our synthesis directly tackles the issue, the inconsistent definitions of GLP-1 RAs across primary studies continue to be a fundamental limitation of the evidence base. Most importantly, a fundamental constraint is that GLP-1 RA use was operationally defined as a pre-surgical prescription in some of the included studies. While our synthesis provides a framework for interpreting this heterogeneity, it cannot overcome the core limitation: we cannot be certain whether the observed associations are due to the drug’s direct perioperative effects or to other factors related to its prior use, such as more structured metabolic care. Consequently, this variability fundamentally obscures the ability to determine an optimal, evidence-based perioperative regimen for GLP-1 RAs in TJA. Third, in our synthesis, we interpreted findings from studies with specific methodological limitations, such as a lack of propensity score matching, with greater caution, as these studies received lower quality scores in our quality assessment. Our overall conclusions are therefore driven by the consistent patterns observed across studies with stronger methodological designs. Fourth, our synthesis faces particular challenges as the substantial overlap in data sources (e.g., multiple studies using PearlDiver) means that the combined sample size overestimates the number of unique patients and may inflate the apparent precision of the evidence. Significant clinical and methodological heterogeneity, including variations in follow-up duration (3 months to 2 years), outcome definitions, and matching strategies, prevented a quantitative meta-analysis, necessitating a narrative synthesis. Fifth, the focus on short- to medium-term outcomes and the exclusion of non-primary and revision procedures limit insights into long-term implant survivorship and safety in more complex surgical scenarios. Furthermore, concentrating solely on U.S. populations may restrict the global relevance of the findings. Finally, by limiting our search to English-language, peer-reviewed publications and excluding grey literature, we might have introduced publication and language bias, potentially overlooking relevant data published in other languages or in non-journal formats.

## Conclusion

In conclusion, this systematic review of observational studies suggests that the use of perioperative GLP-1 receptor agonists has not been consistently associated with an increase in short-term revision rates in patients undergoing total hip and knee arthroplasty. The most notable signal in the data is a potential association with a lower incidence of periprosthetic joint infection (PJI), which appears most pronounced when exposure is defined as active perioperative use. Reported short-term healthcare utilization metrics, such as length of stay and 90-day readmission rates, were generally similar between groups, indicating no consistent negative impact from perioperative GLP-1 RA use. For total shoulder arthroplasty, the evidence remains limited and inconclusive. These findings highlight a need for—but do not yet provide—high-level evidence to guide perioperative management. They underscore the necessity for prospective, controlled studies with standardized exposure definitions and extended follow-up to confirm these associations, assess long-term outcomes, and establish whether a causal perioperative benefit exists.

## Supplementary Information

Supplementary Material 1.

Supplementary Material 2.

## Data Availability

All data generated or analyzed during this study are included in the published article.

## Abbreviations

THA: Total Hip Arthroplasty
TJA: Total Joint Arthroplasty
TKA: Total Knee Arthroplasty
TSA: Total Shoulder Arthroplasty
PRISMA: Preferred Reporting Items for Systematic Reviews and Meta-Analyses
BMI: Body Mass Index
DVT: Deep Vein Thrombosis
VTE: Venous Thromboembolism
ED: Emergency Department
GLP-1 RA: Glucagon-like Peptide-1 Receptor Agonist
LOS: Length of Stay
MI: Myocardial Infarction
NOS: Newcastle-Ottawa Scale
NR: Not Reported
OA: Osteoarthritis
PE: Pulmonary Embolism
PJI: Periprosthetic Joint Infection
Pre-op: Preoperative
Post-op: Postoperative
SD: Standard Deviation
SSI: Surgical Site Infection
T2DM: Type 2 Diabetes Mellitus
AKI: Acute Kidney Injury
CCI: Charlson Comorbidity Index
ECI: Elixhauser Comorbidity Index
HOOS-JR: Hip disability and Osteoarthritis Outcome Score Joint Replacement
