# Supplementary material for: Glucagon-like peptide-1 receptor agonists in total joint arthroplasty: a comprehensive systematic review of what orthopaedic surgeons should know
Source: Arthroplasty. 2026 Mar 23;8:23. doi: 10.1186/s42836-026-00375-w (PMC13007374; doi:10.1186/s42836-026-00375-w)
Supplement: Supplementary file 1 — Supplementary Material 1. [file 42836_2026_375_MOESM1_ESM.docx]

**Supplementary File 1**

**Legends:**

**Table S1.** Search strategy. Details the search strings used for PubMed, Scopus, and Web of Science databases and the number of results yielded from each.

**Table S2.** The Newcastle-Ottawa Scale (NOS) for assessing the quality of non-randomized studies in systematic reviews and meta-analyses. A star (★) denotes that the study met the specific criterion for that item. A dash (—) indicates the criterion was not met. The maximum score is 9 stars.

**Table S3.** Healthcare utilization. Comparison of postoperative outcomes, including operative time, length of hospital stay (LOS), emergency department (ED) visits, revision surgery rates, readmission rates, transfusion rates, and mortality.

**Table S4.** Implant survivorship and local complications. Comparison of local complications, including surgical site infection (SSI), prosthetic joint infection (PJI), wound dehiscence, aseptic loosening, implant dislocation, periprosthetic fractures, and hematoma.

**Table S5.** Systemic adverse events (Embolic and Cardio/Cerebrovascular). Comparison of systemic complications, including venous thromboembolism (VTE), deep vein thrombosis (DVT), pulmonary embolism (PE), and other cardiovascular and cerebrovascular events.

**Table S6.** Systemic adverse events (Metabolic and Infectious). Comparison of systemic complications, including hypoglycemia, anemia, sepsis, and pneumonia.

**Table S7.** Systemic adverse events (Visceral). Comparison of visceral complications, including gastrointestinal, renal, and urinary adverse events.

**Table S1.** Search strategy. Details the search strings used for PubMed, Scopus, and Web of Science databases and the number of results yielded from each.

| **Databaseds** | **Search Strings** | **Number of Records** | **Date of Search** |
| --- | --- | --- | --- |
| **PubMed** | ("Glucagon-Like Peptide-1 Receptor Agonists"[Mesh] OR "Glucagon-Like Peptide-1 Receptor"[Mesh] OR "Glucagon-Like Peptide 1"[Mesh] OR "glucagon-like peptide-1 receptor agonist*"[tiab] OR "GLP-1 receptor agonist*"[tiab] OR "GLP1 receptor agonist*"[tiab] OR "GLP-1 Agonist*"[tiab] OR GLP1[tiab] OR "GLP-1"[tiab] OR "GLP-1RA"[tiab] OR "GLP1RA"[tiab] OR "GLP-1 RA*"[tiab] OR "glucagon-like peptide*"[tiab] OR "Glucose-dependent insulinotropic polypeptide"[tiab] OR "GIP agonist*"[tiab] OR semaglutide[tiab] OR liraglutide[tiab] OR exenatide[tiab] OR dulaglutide[tiab] OR lixisenatide[tiab] OR albiglutide[tiab] OR beinaglutide[tiab] OR loxenatide[tiab] OR tirzepatide[tiab] OR retatrutide[tiab] OR orforglipron[tiab] OR efpeglenatide[tiab] OR "GIP/GLP-1 agonist*"[tiab] OR "twincretin*"[tiab] OR incretin*[tiab])  AND  ("Arthroplasty, Replacement, Hip"[Mesh] OR "Arthroplasty, Replacement, Knee"[Mesh] OR "Arthroplasty, Replacement, Shoulder"[Mesh] OR "Arthroplasty"[Mesh] OR "Joint Prosthesis"[Mesh] OR "knee Arthroplasty*"[tiab] OR "knee replacement*"[tiab] OR "knee reconstruct*"[tiab] OR "knee prosthesis*"[tiab] OR "Artificial knee*"[tiab] OR "hip Arthroplasty*"[tiab] OR "hip replacement*"[tiab] OR "hip reconstruct*"[tiab] OR "hip prosthesis*"[tiab] OR "Artificial hip*"[tiab] OR "hip resurfacing*"[tiab] OR "joint Arthroplasty*"[tiab] OR "joint replacement*"[tiab] OR "joint reconstruct*"[tiab] OR "joint prosthesis*"[tiab] OR "Artificial joint*"[tiab] OR "shoulder Arthroplasty*"[tiab] OR "shoulder replacement*"[tiab] OR "shoulder reconstruct*"[tiab] OR "shoulder prosthesis*"[tiab] OR "Artificial shoulder*"[tiab] OR TKR[tiab] OR TKA[tiab] OR PKR[tiab] OR PKA[tiab] OR THR[tiab] OR PHR[tiab] OR TSR[tiab] OR TSA[tiab]) | **504** | **16 June 2025** |
| **Scopus** | ( TITLE-ABS-KEY ( "glucagon-like peptide-1 receptor agonist*" OR "glp-1 receptor agonist*" OR "glp1 receptor agonist*" OR "glp-1 agonist*" OR glp1 OR "glp-1" OR "glp-1ra" OR glp1ra OR "glp-1 ra*" OR "glucagon-like peptide*" OR "glucose-dependent insulinotropic polypeptide agonist*" OR "gip agonist*" OR semaglutide OR liraglutide OR exenatide OR dulaglutide OR lixisenatide OR albiglutide OR beinaglutide OR loxenatide OR tirzepatide OR retatrutide OR orforglipron OR efpeglenatide OR "gip/glp-1 agonist*" OR "twincretin*" OR incretin* ) )  AND  ( TITLE-ABS-KEY ( "knee arthroplasty*" OR "knee replacement*" OR "knee reconstruct*" OR "knee prosthesis*" OR "artificial knee*" OR "hip arthroplasty*" OR "hip replacement*" OR "hip reconstruct*" OR "hip prosthesis*" OR "artificial hip*" OR "hip resurfacing*" OR "joint arthroplasty*" OR "joint replacement*" OR "joint reconstruct*" OR "joint prosthesis*" OR "artificial joint*" OR "shoulder arthroplasty*" OR "shoulder replacement*" OR "shoulder reconstruct*" OR "shoulder prosthesis*" OR "artificial shoulder*" OR tkr OR tka OR pkr OR pka OR thr OR phr OR tsr OR tsa ) ) | **587** | **16 June 2025** |
| **Web of Science (WOS)** | TS=(("glucagon-like peptide-1 receptor agonist*" OR "GLP-1 receptor agonist*" OR "GLP1 receptor agonist*" OR "GLP-1 Agonist*" OR GLP1 OR "GLP-1" OR "GLP-1RA" OR "GLP1RA" OR "GLP-1 RA*" OR "glucagon-like peptide*" OR "Glucagon receptor agonist*" OR "Glucose-dependent insulinotropic polypeptide agonist*" OR "GIP agonist*" OR semaglutide OR liraglutide OR exenatide OR dulaglutide OR lixisenatide OR albiglutide OR beinaglutide OR loxenatide OR tirzepatide OR retatrutide OR orforglipron OR efpeglenatide OR "GIP/GLP-1 agonist*" OR "twincretin*" OR incretin*)  AND  ("knee Arthroplasty*" OR "knee replacement*" OR "knee reconstruct*" OR "knee prosthesis*" OR "Artificial knee*" OR "hip Arthroplasty*" OR "hip replacement*" OR "hip reconstruct*" OR "hip prosthesis*" OR "Artificial hip*" OR "hip resurfacing*" OR "joint Arthroplasty*" OR "joint replacement*" OR "joint reconstruct*" OR "joint prosthesis*" OR "Artificial joint*" OR "shoulder Arthroplasty*" OR "shoulder replacement*" OR "shoulder reconstruct*" OR "shoulder prosthesis*" OR "Artificial shoulder*" OR TKR OR TKA OR PKR OR PKA OR THR OR PHR OR TSR OR TSA)) | **561** | **16 June 2025** |

**Table S2.** The Newcastle-Ottawa Scale (NOS) for assessing the quality of non-randomized studies in systematic reviews and meta-analyses. A star (★) denotes that the study met the specific criterion for that item. A dash (—) indicates the criterion was not met. The maximum score is 9 stars.

| **Study ID** | **Selection** | | | | **Comparability** | **Outcome** | | | **Total** |
| --- | --- | --- | --- | --- | --- | --- | --- | --- | --- |
|  | **Representativeness of the exposed cohort** | **Selection of the non exposed cohort** | **Ascertainment of exposure** | **Demonstration that outcome of interest was not present at start of study** | **Comparability of cohorts on the basis of the design or analysis** | **Assessment of outcome** | **Was follow-up long enough for outcomes-occur** | **Adequacy of follow up of cohorts (< 20%)** |  |
| Baum et al. 2024 | * | * | * | * | — | * | — | * | 6 |
| Buddhiraju et al. 2024 | * | * | * | * | ** | * | — | * | 8 |
| Choudhury et al. 2025 | * | * | * | * | ** | * | * | * | 9 |
| Elsabbagh et al. 2025 | * | * | * | * | ** | * | * | * | 9 |
| Heo et al. 2024 | * | * | * | * | ** | * | * | * | 9 |
| Katzman et al. 2025 | * | * | * | * | ** | * | * | * | 9 |
| Kim et al. 2024 | * | * | * | * | ** | * | * | * | 9 |
| Kim et al., 2023 | * | * | * | * | ** | * | * | * | 9 |
| Lawand et al. 2025 | * | * | * | * | ** | * | * | * | 9 |
| Levidy et al., 2025 | * | * | * | * | ** | * | * | * | 9 |
| Magruder et al. 2024 | * | * | * | * | ** | * | * | * | 9 |
| Magaldi et al. 2024 | * | * | * | * | ** | * | * | * | 9 |
| Magruder et al., 2023 | * | * | * | * | ** | * | * | * | 9 |
| Seddio et al., 2025 | * | * | * | * | ** | * | — | * | 8 |
| Verhey et al., 2025 | * | * | * | * | ** | * | * | * | 9 |

**Table S3.** Healthcare utilization. Comparison of postoperative outcomes, including operative time, length of hospital stay (LOS), emergency department (ED) visits, revision surgery rates, readmission rates, transfusion rates, and mortality. *Majority of these outcomes are short term (3 months), except Revision Surgery Rate.*

| **Author, Year & Country** | **Operative Time (min) (Mean ± SD)** | | **Length of Hospital Stay LOS (days) (Mean ± SD)** | | **Emergency Department (ED) visits (n/total, %)** | | **Revision Surgery Rate (1/2y, Septic or Aseptic) (n/total, %)** | | **Readmission Rate (n/total, %)** | | **Perioperative HbA1c (Mean ± SD)** | | **Transfusion Rates (n/total, %)** | | **Mortality (n/total, %)** | | |
| --- | --- | --- | --- | --- | --- | --- | --- | --- | --- | --- | --- | --- | --- | --- | --- | --- | --- |
|  | **G** | **C** | **G** | **C** | **G** | **C** | **G** | **C** | **G** | **C** | **G** | **C** | **G** | **C** | **G** | **C** |  |
| TSA |  |  |  |  |  |  |  |  |  |  |  |  |  |  |  |  |  |
| Elsabbagh et al. 2025, USA |  |  | 1.8 ± 1.2 | 1.9 ± 1.8 |  |  | 134/3444 (3.89%) | 482/12,692 (3.80%) | 129/5010 (2.57%) | 528/18,701 (2.82%) |  |  |  |  |  |  |  |
| Lawand et al. 2025, USA |  |  |  |  |  |  | 25/776 (3.2%) | 14/776 (1.8%) | 102/1259 (8.1%) | 66/1259 (5.2%) |  |  | 89/1259 (7.1%) | 54/1259 (4.3%) |  |  |  |
| Seddio et al., 2025, USA |  |  |  |  | 168/632 (26.6%) | 1074/2302 (46.7%) |  |  | 30/632 (4.7%) | 112/2302 (4.9%) |  |  |  |  |  |  |  |
| THA |  |  |  |  |  |  |  |  |  |  |  |  |  |  |  |  |  |
| Buddhiraju et al. 2024 |  |  |  |  | 62/1,044 (5.9%) | 69/1,044 (6.6%) | 18/1,044 (1.7%) | 29/1,044 (2.8%) | 17/1,044 (1.6%) | 21/1,044 (2.0%) | 6.50% | 6.50% |  |  | 10/1,044 (1.0%) | 10/1,044 (1.0%) |  |
| Kim et al. 2024, USA |  |  |  |  |  |  | <11/473 (<2.3%) | 59/1,892 (3.1%) | 53/771 (6.9%) | 300/3,084 (9.7%) |  |  | <11/771 (<1.4%) | 65/3,084 (2.1%) |  |  |  |
| Levidy et al., 2025, USA |  |  |  |  |  |  | 52/2,244 (2.32%) | 51/2,244 (2.27%) |  |  |  |  |  |  |  |  |  |
| Magaldi et al. 2024, USA |  |  | 2.59 ± 0.89 | 2.40 ± 0.80 | 6/66 (9.1%) | 6/126 (4.8%) |  |  | 2/66 (3.0%) | 4/126 (3.2%) |  |  |  |  |  |  |  |
| Magruder et al. 2024, USA |  |  | 2.7 | 2.9 |  |  |  |  | 103/1,653 (6.2%) | 689/7,812 (8.8%) |  |  |  |  |  |  |  |
| Magruder et al. 2024, USA |  |  |  |  |  |  | 30/1,653 (1.8%) | 216/7,812 (2.8%) |  |  |  |  |  |  |  |  |  |
| Verhey et al., 2025, USA |  |  |  |  | 255/5,345 (4.8%) | 310/5,345 (5.8%) | 91/5345 (1.7%) | 92/5345 (1.7%) | 220/5,345 (4.1%) | 241/5,345 (4.5%) |  |  | 40/5,345 (0.7%) | 75/5,345 (1.4%) | 2/5,345 (0.03%) | 5/5,345 (0.1%) |  |
| TKA |  |  |  |  |  |  |  |  |  |  |  |  |  |  |  |  |  |
| Buddhiraju et al. 2024 (TKA), USA |  |  |  |  | 151/2,095 (7.2%) | 161/2,095 (7.7%) | 13/2,095 (0.6%) | 17/2,095 (0.8%) | 23/2,095 (1.1%) | 42/2,095 (2.0%) | 6.50% | 6.40% |  |  | 10/2,095 (0.5%) | 15/2,095 (0.7%) |  |
| Heo et al. 2024, USA |  |  |  |  |  |  | 37/2,388 (1.5%) | 42/2,388 (1.8%) | 168/2,388  (7.0%) | 187/2,388  (7.8%) |  |  |  |  |  |  |  |
| Katzman et al. 2025, USA | 108.4 | 105.5 | 2.1 | 2.5 | 51/865 (5.9%) | 350/8,650 (4.0%) | 20/865 (2.3%) | 224/8,650 (2.6%) | 37/865 (4.3%) | 309/8,650 (3.6%) | 6.50% | 6.30% |  |  |  |  |  |
| Kim et al., 2024, USA |  |  | 2.7 ± 2.3 | 2.9 ± 1.8 |  |  | 58/1,766 (3.3%) | 43/1,766 (2.4%) | 159/2,975 (5.3%) | 266/2,975 (8.9%) |  |  | 20/2,975 (0.7%) | 39/2,975 (1.3%) |  |  |  |
| Levidy et al., 2025, USA |  |  |  |  |  |  | 77/4,700 (1.64%) | 60/4,700 (1.28%) |  |  |  |  |  |  |  |  |  |
| Magruder et al., 2023, USA |  |  | 2.7 | 3.1 |  |  | 281/7,051 (4.0%) | 1,566/34,524 (4.5%) | 491/7,051 (7.0%) | 3,233/34,524 (9.4%) |  |  |  |  |  |  |  |

**Table S4.** Implant survivorship and local complications. Comparison of local complications, including surgical site infection (SSI), prosthetic joint infection (PJI), wound dehiscence, aseptic loosening, implant dislocation, periprosthetic fractures, and hematoma.

| **Author, Year & Country** | **SSI (n/total, %)** | | **Superficial SSI (n/total, %)** | | **Deep SSI /PJI (n/total, %)** | | **Wound Dehiscence (n/total, %)** | | **Aseptic Loosing (n/total, %)** | | **Implant Dislocation (n/total, %)** | | | **Periprosthetic Fractures (n/total, %)** | | **Hematoma (n/total, %)** | |
| --- | --- | --- | --- | --- | --- | --- | --- | --- | --- | --- | --- | --- | --- | --- | --- | --- | --- |
|  | **G** | **C** | **G** | **C** | **G** | **C** | **G** | **C** | **G** | **C** | **G** | **C** | **G** | | **C** | **G** | **C** |
| TSA |  |  |  |  |  |  |  |  |  |  |  |  |  | |  |  |  |
| Elsabbagh et al. 2025, USA (3m) | 16/5,010 (0.32%) | 55/18,701 (0.29%) |  |  |  |  |  |  |  |  |  |  |  | |  |  |  |
| Elsabbagh et al. 2025, USA (2y) |  |  |  |  | 15/3,444 (0.44%) | 45/12,692 (0.35%) |  |  | 119/3,444 (3.46%) | 437/12,692 (3.44%) |  |  | 111/3,444 (3.22%) | | 497/12,692 (3.92%) |  |  |
| Lawand et al. 2025, USA (2y) |  |  |  |  | <10 | <10 |  |  | <10 | <10 |  |  | <10 | | <10 |  |  |
| Seddio et al., 2025, USA (3m) | <11/632 (<1.7%) | 69/2,302 (3.0%) |  |  |  |  | <11/632 (<1.7%) | 80/2,302 (3.5%) |  |  | 17/632 (2.7%) | 91/2,302 (4.0%) |  | |  |  |  |
| TKA |  |  |  |  |  |  |  |  |  |  |  |  |  | |  |  |  |
| Magruder et al. 2023, USA (2y) |  |  |  |  | 151/7,051 (2.1%) | 1,029/34,524 (3.0%) |  |  | 0/7,051 (0.0%) | 17/34,524 (0.0%) |  |  | 0/7,051 (0.0%) | | 40/34,524 (0.1%) |  |  |
| Buddhiraju et al. 2024, USA (3m) |  |  | 13/2,095 (0.6%) | 10/2,095 (0.5%) | 23/2,095 (1.1%) | 23/2,095 (1.1%) |  |  |  |  |  |  |  | |  |  |  |
| Heo et al. 2024, USA (3m) | 112/2,388 (4.7%) | 108/2,388 (4.5%) |  |  | 53/2,388 (2.2%) | 55/2,388 (2.3%) | 34/2,388 (1.4%) | 38/2,388 (1.6%) |  |  |  |  | 3/2,388 (0.1%) | | 1/2,388 (0.1%) |  |  |
| Heo et al. 2024, USA (1y) |  |  |  |  | 81/2,388 (3.4%) | 76/2,388 (3.2%) |  |  | 24/2,388 (1.0%) | 27/2,388 (1.1%) |  |  | 4/2,388 (0.2%) | | 2/2,388 (0.1%) |  |  |
| Katzman et al. 2025, USA (2y) |  |  |  |  | 10/865 (1.2%) | 115/8,650 (1.3%) |  |  | 10/865 (1.2%) | 109/8,650 (1.3%) |  |  |  | |  |  |  |
| Kim et al., 2024 (3m), USA |  |  |  |  | 31/2,975 (1.0%) | 55/2,975 (1.8%) | 42/2,975 (1.4%) | 63/2,975 (2.1%) | 11/2,975 (0.4%) | <11/2,975 (<0.4%) |  |  | <11/2,975 (<0.4%) | | <11/2,975 (<0.4%) | <11 / 2,975 (<0.4%) | <11 / 2,975 (<0.4%) |
| Kim et al., 2024 (2y), USA |  |  |  |  | 51/1,766 (2.9%) | 53/1,766 (3.0%) |  |  | 27/1,766 (1.5%) | 16/1,766 (0.9%) |  |  | <11/1,766 (<0.6%) | | <11/1,766 (<0.6%) |  |  |
| Levidy et al., 2025, USA (3m) |  |  |  |  | 44/4,700 (0.94%) | 68/4,700 (1.45%) |  |  |  |  |  |  | 22/4,700 (0.47%) | | 10/4,700 (0.21%) |  |  |
| Levidy et al., 2025, USA (1y) |  |  |  |  | 57/4,700 (1.21%) | 96/4,700 (2.04%) |  |  |  |  |  |  | 33/4,700 (0.70%) | | 16/4,700 (0.34%) |  |  |
| Magruder et al., 2023, USA (2y) |  |  |  |  | 151/7,051 (2.1%) | 1,029/34,524 (3.0%) |  |  | 0/7,051 (0.0%) | 17/34,524 (0.0%) |  |  | 0/7,051 (0.0%) | | 40/34,524 (0.1%) |  |  |
| THA |  |  |  |  |  |  |  |  |  |  |  |  |  | |  |  |  |
| Buddhiraju et al. 2024, USA (3m) |  |  | 10/1,044 (1.0%) | 10/1,044 (1.0%) | 22/1,044 (2.1%) | 38/1,044 (3.6%) |  |  |  |  |  |  |  | |  |  |  |
| Kim et al. 2024, USA (3m) |  |  |  |  | 12/771 (1.6%) | 100/3,084 (3.2%) | 18/771 (2.3%) | 70/3,084 (2.3%) | 0/771 (0.0%) | <11/3,084 (<0.4%) | 12/771 (1.6%) | 53/3,084 (1.7%) | <11/771 (<1.4%) | | 28/3,084 (0.9%) | 0 / 771 (0.0%) | 39 / 3,084 (1.3%) |
| Kim et al. 2024, USA (2y) |  |  |  |  | <11/473 (<2.3%) | 58/1,892 (3.1%) |  |  | 0/473 (0.0%) | <11/1,892 (<0.6%) | <11/473 (<2.3%) | 26/1,892 (1.4%) | <11/473 (<2.3%) | | 15/1,892 (0.8%) |  |  |
| Levidy et al., 2025, USA (3m) |  |  |  |  | 34/2,244 (1.52%) | 47/2,244 (2.09%) |  |  |  |  |  |  | 26/2,244 (1.16%) | | 27/2,244 (1.20%) |  |  |
| Levidy et al., 2025, USA (1y) |  |  |  |  | 48/2,244 (2.14%) | 63/2,244 (2.81%) |  |  |  |  |  |  | 39/2,244 (1.74%) | | 31/2,244 (1.38%) |  |  |
| Magruder et al. 2024, USA (3m) | 17/1,653 (1.0%) | 106/7,812 (1.4%) |  |  |  |  |  |  |  |  |  |  |  | |  |  |  |
| Magruder et al. 2024, USA (2y) |  |  |  |  | 27/1,653 (1.6%) | 223/7,812 (2.9%) |  |  | 0/1,653 (0.0%) | 0/7,812 (0.0%) |  |  | 0/1,653 (0.0%) | | 39/7,812 (0.5%) |  |  |
| Verhey et al., 2025, USA (3m) | 34/5,345 (0.6%) | 22/5,345 (0.4%) |  |  | 64/5,345 (1.2%) | 59/5,345 (1.1%) | 52/5,345 (1.0%) | 41/5,345 (0.8%) | 16/5,345 (0.3%) | 16/5,345 (0.3%) | 43/5,345 (0.8%) | 38/5,345 (0.7%) | 3/5,345 (0.06%) | | 6/5,345 (0.1%) | 16 / 5,213 (0.3%) | 23 / 5,213 (0.4%) |
| Verhey et al., 2025, USA (2y) | 44/5,345 (0.8%) | 30/5,345 (0.6%) |  |  | 106/5,345 (2.0%) | 91/5,345 (1.7%) |  |  | 38/5,345 (0.7%) | 36/5,345 (0.7%) | 65/5,345 (1.2%) | 60/5,345 (1.1%) | 4/5,345 (0.07%) | | 6/5,345 (0.1%) |  |  |

*Baum et al., 2024 and Choudhury et al. 2025 studies didn’t report local complications*

SSI: Surgical Site Infection, PJI: Prosthetic Joint Infection, *G: Glucagon-like peptide-1, C: Control, THA: Total Hip Arthroplasty, TKA: Total Knee Arthroplasty, TSA: Total Shoulder Arthroplasty, y: Year, m: Month, USA: United States of America*

**Table S5.** Systemic adverse events (Embolic and Cardio/Cerebrovascular). Comparison of systemic complications, including venous thromboembolism (VTE), deep vein thrombosis (DVT), pulmonary embolism (PE), and other cardiovascular and cerebrovascular events

| **Author, Year & Country** | **VTE (n/total, %)** | | **DVT (n/total, %)** | | **PE (n/total, %)** | | **Cardiovascular Events (n/total, %)** | | **Cerebrovascular Events (n/total, %)** | |
| --- | --- | --- | --- | --- | --- | --- | --- | --- | --- | --- |
|  | **G** | **C** | **G** | **C** | **G** | **C** | **G** | **C** | **G** | **C** |
| TSA |  |  |  |  |  |  |  |  |  |  |
| Elsabbagh et al. 2025, USA (3m) |  |  | 30/5,010 (0.60%) | 86/18,701 (0.46%) | 14/5,010 (0.28%) | 71/18,701 (0.38%) |  |  |  |  |
| Lawand et al. 2025, USA (3m) |  |  | 32/1,259 (1.6%) | 11/1,259 (0.9%) | 26/1,259 (1.7%) | 16/1,259 (1.3%) | 28/1,259 (1.6%) | 10/1,259 (0.9%) | 37/1,259 (2.9%) | 18/1,259 (1.4%) |
| Seddio et al., 2025, USA (3m)* | 12/632 (1.9%) | 114/2,302 (5.0%) |  |  |  |  | <11/632 (<1.7%) | 84/2,302 (3.6%) |  |  |
| THA |  |  |  |  |  |  |  |  |  |  |
| Buddhiraju et al. 2024, USA (3m) |  |  | 10/1,044 (1.0%) | 11/1,044 (1.1%) | 10/1,044 (1.0%) | 10/1,044 (1.0%) |  |  |  |  |
| Kim et al. 2024, USA (3m) |  |  | <11/771 (<1.4%) | 36/3,084 (1.2%) | <11/771 (<1.4%) | <11/771 (<1.4%) |  |  |  |  |
| Magruder et al. 2024, USA (3m) | 11/1,653 (0.7%) | 70/7,812 (0.9%) | 0/1,653 (0.0%) | 55/7,812 (0.7%) | 0/1,653 (0.0%) | 34/7,812 (0.4%) | 0/1,653 (0.0%) | 55/7,812 (0.7%) | 0/1,653 (0.0%) | 68/7,812 (0.9%) |
| Verhey et al., 2025, USA (3m) |  |  | 26/5,345 (0.5%) | 30/5,345 (0.6%) | 8/5,345 (0.1%) | 9/5,345 (0.2%) | 5/5,345 (0.09%) | 3/5,345 (0.06% | 1/5,345 (0.01%) | 0/5,345 (0.0%) |
| TKA |  |  |  |  |  |  |  |  |  |  |
| Magruder et al. 2023, USA (3m) | 80/7,051 (1.1%) | 353/34,524 (1.0%) | 58/7,051 (0.8%) | 188/34,524 (0.5%) | 36/7,051 (0.5%) | 214 /34,524 (0.6%) | 70 /7,051 (1.0%) | 229 /34,524 (0.7%) | 85 /7,051 (1.2%) | 304 /34,524 (0.9%) |
| Buddhiraju et al. 2024, USA (3m) |  |  | 15/2,095 (0.7%) | 21/2,095 (1.0%) | 10/2,095 (0.5%) | 13/2,095 (0.6%) |  |  |  |  |
| Heo et al. 2024, USA (3m) |  |  | 39/2,388 (1.6%) | 45/2,388 (1.9%) |  |  | 3/2,388 (0.1%) | 4/2,388 (0.2%) | 10/2,388 (0.4%) | 18/2,388 (0.8%) |
| Kim et al., 2024, USA (3m) |  |  | 30/2,975 (1.0%) | 34/2,975 (1.1%) | <11/2,975 (<0.4%) | 17/2,975 (0.6%) | <11/2,975 (<0.4%) | <11/2,975 (<0.4%) |  |  |

**In Seddio et al., 2025; VTE included PE and DVT, while in other studies it does not.*

*VTE: Venous Thromboembolism, DVT: Deep Vein Thrombosis, PE: Pulmonary Embolism, G: Glucagon-like peptide-1, C: Control,, THA: Total Hip Arthroplasty, TKA: Total Knee Arthroplasty, TSA: Total Shoulder Arthroplasty, y: Year, m: Month, USA: United States of America*

**Table S6.** Systemic adverse events (Metabolic and Infectious). Comparison of systemic complications, including hypoglycemia, anemia, sepsis, and pneumonia.

| **Author, Year & Country** | **Hypoglycemia (n/total, %)** | | **Anemia (n/total, %)** | | **Sepsis (n/total, %)** | | **Pneumonia (n/total, %)** | |
| --- | --- | --- | --- | --- | --- | --- | --- | --- |
|  | **G** | **C** | **G** | **C** | **G** | **C** | **G** | **C** |
| TKA |  |  |  |  |  |  |  |  |
| Magruder et al., 2023 (3m), USA | 134/7,051 (1.9%) | 421/34,524 (1.2%) |  |  | 0/7,051 (0.0%) | 124/34,524 (0.4%) | 195/7,051(2.8%) | 571/34,524 (1.7%) |
| Buddhiraju et al. 2024 (3m), USA |  |  |  |  |  |  | <10/2,095 (0.5%)* | <10/2,095 (0.5%) |
| Heo et al. 2024 (3m), USA | 45/2,388 (1.9%) | 35/2,388 (1.5%) |  |  |  |  | 37/2,388 (1.5%) | 38/2,388 (1.6%) |
| THA |  |  |  |  |  |  |  |  |
| Magruder et al. 2024 (3m), USA | 0/1,653 (0.0%) | 81/7,812 (1%) |  |  | 0/1,653 (0.0%) | 33/7,812 (0.4%) | 32/1,653(1.9%) | 111/7,812(1.4% ) |
| Verhey et al., 2025 (3m), USA | 0/5,345 (0.0%) | 1/5,345 (0.0%) | 23/5,345 (0.4%) | 40/5,345 (0.7%) | 15/5,345 (0.3%) | 15/5,345 (0.3%) | 21/5,345 (0.39%) | 18/5,345 (0.3%) |
| TSA |  |  |  |  |  |  |  |  |
| Elsabbagh et al. 2025 (3m), USA | 8/5,010 (0.2%) | 47/18,701 (0.3%) | DA:742/5010 (14.8%)  BLA:163/5010 (3.3%) | DA:2993/18701 (16.0%)  BLA:677/18701 (3.6%) | 29/5,010 (0.6%) | 149/18,701 (0.8%) | 69/5010 (1.38%) | 266/18,701 (1.42%) |
| Lawand et al. 2025, USA |  |  | 166/1,259 (13.2%) | 128/1,259 (10.2%) |  |  | 42/1,259 (3.3%) | 19/1,259 (1.5%) |
| Seddio et al., 2025, USA |  |  |  |  | 20/632 (3.2%) | 135/2,302 (5.9%) | 19/632 (3%) | 240/2,302 (10.4%) |

*Aspiration Pneumonia

**Table S7.** Systemic adverse events (Visceral). Comparison of visceral complications, including gastrointestinal, renal, and urinary adverse events.

| **Author, Year & Country** | **Constipation (n/total, %)** | | **Diarrhea (n/total, %)** | | **Nausea or Vomiting (n/total, %)** | | **Pancreatitis (n/total, %)** | | **Bowel Obstruction (n/total, %)** | | **Cholecystitis (n/total, %)** | | **Acute Kidney Injury (n/total, %)** | | **Urinary Tract Infection (n/total, %)** | | **Urinary Retention (n/total, %)** | |
| --- | --- | --- | --- | --- | --- | --- | --- | --- | --- | --- | --- | --- | --- | --- | --- | --- | --- | --- |
|  | **G** | **C** | **G** | **C** | **G** | **C** | **G** | **C** | **G** | **C** | **G** | **C** | **G** | **C** | **G** | **C** | **G** | **C** |
| TKA |  |  |  |  |  |  |  |  |  |  |  |  |  |  |  |  |  |  |
| Baum et al. 2024, USA (30d) | 80/1,876 (4.3%) | 6,611/150,099 (4.4%) | 39/1,876 (2.1%) | 2,458/150,099 (1.6%) |  |  |  |  |  |  |  |  |  |  |  |  |  |  |
| Baum et al. 2024, USA (3m) | 80/1,876 (4.3%) | 6,611/150,099 (4.4%) | 39/1,876 (2.1%) | 2,458/150,099 (1.6%) | 83/1,876 (4.4%) | 5,290/150,099 (3.5%) | 0/1,876 (0.0%) | 195/150,099 (0.1%) | 0/1,876 (0.0%) | 331/150,099 (0.2%) | 0/1,876 (0.0%) | 275/150,099 (0.2%) |  |  |  |  |  |  |
| Buddhiraju et al. 2024 (TKA), USA |  |  |  |  |  |  |  |  |  |  |  |  | 46/2,095 (2.2%) | 44/2,095 (2.1%) |  |  |  |  |
| Heo et al. 2024 (3m), USA |  |  |  |  |  |  |  |  |  |  |  |  | 84/2,388 (3.5%) | 90/2,388 (3.8%) | 150/2,388 (6.3%) | 151/2,388 (6.3%) |  |  |
| THA |  |  |  |  |  |  |  |  |  |  |  |  |  |  |  |  |  |  |
| Baum et al. 2025, USA (3m) | 25/667 (3.7%) | 3396/83,587 (4.1%) | 11/667 (1.6%) | 1150/83,587 (1.4%) | 19/667 (2.8%) | 2271/83,587 (2.7%) | 0/667 (0.0%) | 101/83,587 (0.1%) | 0/667 (0.0%) | 236/83,587 (0.3%) | 0/667 (0.0%) | 161/83,587 (0.2%) |  |  |  |  |  |  |
| Buddhiraju et al. 2024 |  |  |  |  |  |  |  |  |  |  |  |  | 21/1,044 (2.0%) | 15/1,044 (1.4%) |  |  |  |  |
| Kim et al. 2024 (3m), USA |  |  |  |  |  |  |  |  |  |  |  |  | 25/771 (3.2%) | 134/3,084 (4.3%) | 28/771 (3.6%) | 152/3,084 (4.9%) |  |  |
| Magaldi et al. 2024, USA |  |  |  |  | 12/66 (18.2%) | 8/126 (6.0%) |  |  |  |  |  |  |  |  |  |  | 7/66 (10.6%) | 10/126 (8.0%) |
| Magruder et al. 2024 (3m), USA |  |  |  |  |  |  |  |  |  |  |  |  | 46/1,653 (2.8%) | 301/7,812 (3.9%) |  |  |  |  |
| Verhey et al., 2025 (3m), USA |  |  |  |  |  |  |  |  |  |  |  |  | 26/5,345 (0.5%) | 30/5,345 (0.6%) |  |  |  |  |
| TSA |  |  |  |  |  |  |  |  |  |  |  |  |  |  |  |  |  |  |
| Elsabbagh et al. 2025 (3m), USA |  |  |  |  |  |  |  |  |  |  |  |  |  |  | 156/5,010 (3.1%) | 607/18,701 (3.3%) |  |  |
| Lawand et al. 2025, USA |  |  |  |  |  |  |  |  |  |  |  |  | 58/1,259 (4.6%) | 34/1,259 (2.7%) |  |  |  |  |
| Seddio et al., 2025, USA |  |  |  |  |  |  |  |  |  |  |  |  | 34/632 (5.4%) | 269/2,302 (11.7%) | 53/632 (8.4%) | 482/2,302 (20.9%) |  |  |
